# The effects of selected neglected tropical diseases on economic performance at the macrolevel in Africa

**DOI:** 10.1186/s12879-024-09302-3

**Published:** 2024-05-02

**Authors:** Mustapha Immurana, Kwame Godsway Kisseih, Ibrahim Abdullahi, Muniru Azuug, Alfred Kwesi Manyeh, Ayisha Mohammed, Toby Joseph Mathew Kizhakkekara

**Affiliations:** 1 Institute of Health Research, University of Health and Allied Sciences, Ho, Ghana; 2 Christian Health Association of Ghana Secretariat, Accra, Ghana; 3 Vignan’s Foundation for Science, Technology & Research, Guntur, India; 4 Safe Haven Insurance Brokers Ltd, Accra, Ghana; 5 Department of Economics Education, University of Education, Winneba, Ghana; 6 Akenten Appiah-Menka University of Skills Training and Entrepreneurial Development, Kumasi, Ghana; 7 PG Department of Economics, EKNM Government College Elerithattu, Elerithattu (PO), Kasaragod District, Kerala, 671314 India

**Keywords:** Neglected tropical diseases, Economic performance, Africa

## Abstract

**Background:**

Neglected tropical diseases (NTDs) such as leprosy, lymphatic filariasis (LF), schistosomiasis and onchocerciasis are endemic in several African countries. These diseases can lead to severe pain and permanent disability, which can negatively affect the economic productivity of the affected person(s), and hence resulting into low economic performance at the macrolevel. Nonetheless, empirical evidence of the effects of these NTDs on economic performance at the macrolevel is sparse. This study therefore investigates the effects of the above-mentioned NTDs on economic performance at the macrolevel in Africa.

**Methods:**

The study employs a panel design with data comprising 24 to 45 African countries depending on the NTD in question, over the period, 2002 to 2019. Gross domestic product (GDP) is used as the proxy for economic performance (Dependent variable) and the prevalence of the above-mentioned NTDs are used as the main independent variables. The random effects (RE), fixed effects (FE) and the instrumental variable fixed effects (IVFE) panel data regressions are used as estimation techniques.

**Results:**

We find that, an increase in the prevalence of the selected NTDs is associated with a fall in economic performance in the selected African countries, irrespective of the estimation technique used. Specifically, using the IVFE regression estimates, we find that a percentage increase in the prevalence of leprosy, LF, schistosomiasis and onchocerciasis is associated with a reduction in economic performance by 0.43%, 0.24%, 0.28% and 0.36% respectively, at either 1% or 5% level of significance.

**Conclusion:**

The findings highlight the need to increase attention and bolster integrated efforts or measures towards tackling these diseases in order to curb their deleterious effects on economic performance. Such measures can include effective mass drug administration (MDA), enhancing access to basic drinking water and sanitation among others.

**Supplementary Information:**

The online version contains supplementary material available at 10.1186/s12879-024-09302-3.

## Introduction

Neglected tropical diseases (NTDs) are a group of poverty diseases that affect over 1 billion people in the world [1]. In Africa, leprosy, lymphatic filariasis (LF), schistosomiasis and onchocerciasis are some of the common NTDs. For leprosy, if left untreated, it can lead to gradual and permanent disabilities [2]. LF is associated with unusual expansion of parts of the body and this normally comes with pain and severe disability [3]. With regard to schistosomiasis, it affects close to 240 million people in the world [4], and not less than 90% of those requiring treatment for it live in Africa [5]. Schistosomiasis can lead to painful urination, lower abdominal pain, tiredness among others [6]. In young girls and women, if left untreated, schistosomiasis can lead to female genital schistosomiasis (FGS). Some of the effects of FGS include miscarriage, infertility and a three-fold higher risk of HIV infection [6, 7]. With regard to onchocerciasis, it can lead to disfiguring conditions of the skin, severe itching as well as permanent blindness, and more than 99% of the people infected live in Africa [8]. 

The deleterious effects of these diseases including severe pain and disability, tend to prevent or reduce the participation of affected persons (and their caregivers) in economic activities. For instance, among households in Cameroon, it has been found that, the annual average working days lost due to leprosy were 115 days [9]. This can negatively affect economic performance at the macrolevel, especially in Africa, where these NTDs are endemic.

Nonetheless, there is a dearth of empirical evidence of the effects of these NTDs on economic performance in the African context, with the few available either focusing on the microlevel (individuals or households) and one NTD in a single country [10–15] or one NTD across African or other countries [16–19]. However, relative to the microlevel, knowing the effects of these NTDs on economic performance at the macrolevel across several African countries is more important towards drawing or increasing attention on the need to bolster efforts geared at tackling these diseases. This is because, the studies focusing on the microlevel were not nationwide in nature, hence were likely to have underestimated the economic effects of these NTDs since they did not capture all affected individuals or households in a particular country. Moreover, among the studies that provided macrolevel evidence across African countries, none of them focused on more than one NTD, although providing evidence of the economic effects of a number of NTDs helps in revealing the need to enhance integrated efforts towards tackling these diseases. Specifically, the study by Wright [16] investigated the effect of schistosomiasis on gross domestic product (GDP) and Mathew et al. [17] examined the effect of LF on GDP. Similarly, Marques et al. [19] and Kim et al. [18] assessed the economic effects of blindness and vision impairment (not disease specific)^1^, as well as onchocerciasis (river blindness), respectively. In addition, the study by Mathew et al. [17] focused on periods prior to mass drug administration (MDA). However, knowing the economic effects of these NTDs in the era of MDA is very important in unearthing the need to increase current efforts towards fighting these diseases. Also, to the best of our knowledge, no study has provided macrolevel evidence of the effect of leprosy on economic performance in Africa.

To this end, this study investigates the effects of leprosy, LF, schistosomiasis and onchocerciasis on economic performance at the macrolevel across African countries, from 2002 to 2019. Thus, this study, to the best of our knowledge, is the first to provide empirical evidence of the effects of more than one NTD (including leprosy) on economic performance at the macrolevel in Africa. Doing so helps in unearthing the magnitude of the current effects of these diseases on economic performance across African countries, which is important towards increasing attention on the need to fight these diseases through integrated efforts. These integrated approaches, will aid in achieving the United Nations' [20] sustainable development goal (SDG) 3.3 target of ending the epidemics of NTDs by 2030.

## Methods

### Study design, data and variables

This study employs a panel design made up of annual data on 24 to 45 African countries (see Table 1) depending on the NTD in question, over the period 2002 to 2019. The study period and the number of countries are largely dictated by data availability on all variables, as well as the need to provide evidence of the economic effects of NTDs during the MDA era. The dependent variable is economic performance (EP), proxied by GDP measured in constant 2015 US Dollars($), while the point prevalence of leprosy, LF, schistosomiasis and onchocerciasis measured in percentages are the main independent variables used. Net inflows of foreign direct investment (FDI), inflation (I (GDP deflator)), regulatory quality (R (perceptions on the capability of government to institute concrete regulations and policies that enhance the development of the private sector)), domestic investment (D (gross fixed capital formation)), expenditure (E (both household and government consumption expenditure)), imports (IM) and exports (EX) are used as control variables. For some of the control variables above, the information in parentheses provided after their respective notations represent their definitions or proxies. FDI, domestic investment, consumption expenditure, imports and exports are all measured as percentages of GDP. Inflation is measured in percentages, while regulatory quality is measured on a score of -2.5 to 2.5. These control variables are selected based on literature [21–25]. The data on the NTDs are obtained from the website of the Global Burden of Diseases Study [26], data on regulatory quality are obtained from the World Bank’s World-Wide Governance Indicators [27] and the data on all the remaining variables are obtained from the World Bank’s World Development Indicators [28]. Summary statistics of the variables can be found in the appendix (supplementary material).

**Table 1:** Average prevalence of NTDs per-country, 2002–2019

Country	Leprosy	Lymphatic Filariasis	Schistosomiasis	Onchocerciasis
Algeria			0.0080325	
Angola	0.0001408	0.0299647	0.0814061	0.0062795
Benin	0.0000851	0.0081071	0.2723303	0.0067721
Botswana	0.0000114		0.1153732	
Burkina Faso	0.0000848	0.094867	0.0977698	0.0000625
Burundi	0.0001331		0.2873656	0.0278768
Cabo Verde	0.000019			
Cameroon	0.0000312	0.02163	0.1595189	0.0619883
Central African Republic	0.000773	0.1151345	0.0803251	0.0771679
Chad	0.0001525	0.0328954	0.0688049	0.0192785
Congo, Dem. Rep.	0.0001988	0.060898	0.1230586	0.1378723
Congo, Rep.	0.0000777	0.047308	0.1352105	0.0093404
Cote d’Ivoire	0.0000906	0.1488467	0.2170407	0.0000739
Djibouti	0.0000471		0.0020977	
Egypt, Arab Rep.	0.0000201	0.0106238	0.1199214	
Equatorial Guinea	0.0000274	0.0376968	0.0409145	0.0313065
Eritrea	0.0000621	0.005628	0.1103604	
Ethiopia	0.0001351	0.0102572	0.1895536	0.0068153
Gabon	0.0000435	0.0263702	0.2544782	
Gambia, The	0.0000552	0.0053959	0.2897632	
Ghana	0.0000242	0.0239514	0.2359097	0.0009013
Guinea	0.0001665	0.0200198	0.1494091	0.0050223
Guinea-Bissau	0.0001837	0.0424292	0.1255006	0.0000547
Kenya	0.0000175	0.0653431	0.1977624	
Lesotho	0.0000714			
Libya	4.25e-06		0.1371126	
Madagascar	0.0001806	0.0560587	0.099381	
Mali	0.0000487	0.1115966	0.1449072	0.0000531
Mauritania	0.0000504		0.0573097	
Mauritius	0.0000151		0.3789404	
Morocco	0.0000169		0.0187071	
Mozambique	0.0002369	0.1413271	0.0863711	
Niger	0.000087	0.0673039	0.0513824	0.0000354
Nigeria	0.0000794	0.0684811	0.185798	0.0183808
Rwanda	0.0000326		0.2597715	
Senegal	0.0000642	0.0264766	0.2508055	0.0000423
Seychelles	0.0000635			
Sierra Leone	0.000142	0.0947469	0.1019397	0.0471933
South Africa	7.04e-06		0.0836127	
Sudan	0.0001145	0.0216371	0.0269053	0.003327
Tanzania	0.0000747	0.0772336	0.1890059	0.0063653
Togo	0.0000589	0.0062224	0.1383933	0.0010697
Tunisia	4.82e-06		0.0199197	
Uganda	0.0000352	0.0285715	0.1868933	0.0111456
Zambia	0.0000368	0.0665192	0.162095	
Zimbabwe	0.0000465	0.0179026	0.1083517	

As regards the expected signs of the variables, as indicated in the previous section (Introduction), we expect all the NTDs to have a negative effect on economic performance. This is because, these NTDs are associated with severe morbidity and disability which can reduce the economic productivity of the affected persons, hence hindering economic performance. Turning to the control variables, the effect of FDI on economic performance is uncertain. This is because, FDI can be detrimental to economic performance by crowding out domestic investment [22, 29], while on the other hand, FDI can be associated with higher factor productivity in the recipient country, hence, enhancing economic performance [30]. Similarly, the sign of inflation is uncertain [21, 23–25, 31]. This is because, while rising prices can reduce the ability of producers to purchase production inputs, which could reduce economic performance, conversely, rising prices could imply more demand for relatively fewer goods and services, which would result in higher revenues as well as willingness on the part of firms to produce more goods and services, leading to higher economic performance.

We expect regulatory quality to have a positive effect on economic performance since rising regulatory quality has the potential to enhance private sector development. Consumption expenditure and domestic investment are expected to have negative and positive effects on economic performance since they represent leakages and injections in productive capacity, respectively [21–23]. The sign of imports is uncertain. This is because, if imports are made up of capital goods, they can be used to expand production which would enhance economic performance. Nonetheless, if imports are made up of consumption goods, the goods will not contribute towards investment in domestic production, hence leading to low economic performance [22]. Last but not the least, the expected sign of exports is positive since higher exports imply more domestic production as well as foreign exchange that can be reinvested in local production.

### Model and estimation techniques

To examine the effects of the selected NTDs on economic performance, we specify the following equation:

1 $$\eqalign{ E{P_{it}}\, = \, & {\varpi _0}\, + \,{\varpi _1}\,NT{D_{it}}\, + \,{\varpi _2}\,FD{I_{it}}\, + \,{\varpi _3}\,{I_{it}}\, + \,{\varpi _4}\,{R_{it}} \cr & + \,{\varpi _5}\,{D_{it}}\, + \,{\varpi _6}\,{E_{it}}\, + \,{\varpi _7}\,E{X_{it}}\, + \,{\varpi _8}\,I{M_{it}}\, + {\varepsilon _{it}} \cr} $$

where t refers to the years, i represents the countries and $\varvec{{\upepsilon }}$ is the error term. NTD is a vector for the prevalence of leprosy, LF, schistosomiasis and onchocerciasis while ${\varpi }_{0}$ is the intercept of the equation and the rest of the ${\varpi }_{s}$ are coefficients of their respective variables. Models are run separately for each of the selected NTDs. All other notations are as defined in the previous sub-section.

Regarding the estimation technique, given the panel nature of our data, we employ the panel random effects (RE) and fixed effects (FE) regressions as our baseline estimation techniques. We use cluster robust standard errors to deal with any possible serial correlation and heteroscedasticity in all our estimates. It must, however be stressed that, the Sargan-Hansen test of overidentifying restrictions (available upon request) show the FE regression as the preferred technique or approach but results of both approaches are reported.

Nonetheless, in examining the effects of NTDs on economic performance, one estimation problem that is likely to happen is endogeneity. Thus, the likelihood that, the dependent variable; economic performance can affect NTDs, which if not dealt with can lead to biased estimates. This is because, our economic performance indicator (i.e GDP) is normally used to represent income. Thus, since NTDs are diseases of poverty [1, 32], people with higher income are less likely to experience these diseases and vice versa. Given this, and the results of the Sargan-Hansen test of overidentifying restrictions stated above, we use the instrumental variable fixed effects (IVFE) regression as a third estimator in order to deal with any potential endogeneity as well as for robustness purposes.

In using the IVFE regression, we employ the first lag of the respective NTD and the first lag of gross national expenditure as instruments. Thus, while the previous year’s values of both NTDs prevalence and gross national expenditure can affect the current levels of NTDs prevalence, they (instruments) are less likely to be affected by the current level of economic performance. The data on gross national expenditure are obtained from the World Bank’s World Development Indicators [28].

We use the Kleibergen-Paap rk Wald F test for weak identification (WI stat.), the Kleibergen-Paap rk LM under-identification test (Id stat.), and the Hansen overidentification test (Hansen *j* stat.) to examine the appropriateness of our IVFE regression estimates. Thus, the insignificance and significance of the p-values of the Hansen *j* stat. and Id stat. respectively, as well as the WI stat. value being greater than the Stock-Yogo test critical values (not reported for brevity), confirm the suitability of the IVFE regression estimates [33–38].

In our regression analysis, logarithms (log) of all variables (except FDI, inflation and regulatory quality) are used in order to reduce the differences in the units of measurement of variables as well as facilitate the interpretation of results as elasticities [39, 40]. We do not take logarithms of FDI, inflation and regulatory quality because they have negative values.

## Results

This section presents results of the average prevalence of the selected NTDs per-country over the study period, the trends of the selected NTDs per-country, as well as the regression estimates of the effects of the selected NTDs on economic performance in the sampled African countries.

### Average prevalence of NTDs per-country, 2002–2019

Table 1 shows the average prevalence of the selected NTDs per-country, over the study period. In general, relative to the other NTDs, the prevalence of leprosy is low. Cote d’Ivoire has the highest prevalence of LF (0.15%) while Mauritius has the highest prevalence of schistosomiasis (0.38%). The highest prevalence of onchocerciasis (0.14%) is found in the Democratic Republic of Congo. Details of the average prevalence of the selected NTDs for the other countries can be found in Table 1.

### Trends of selected NTDs per-country, 2002–2019

**Fig. 1:**
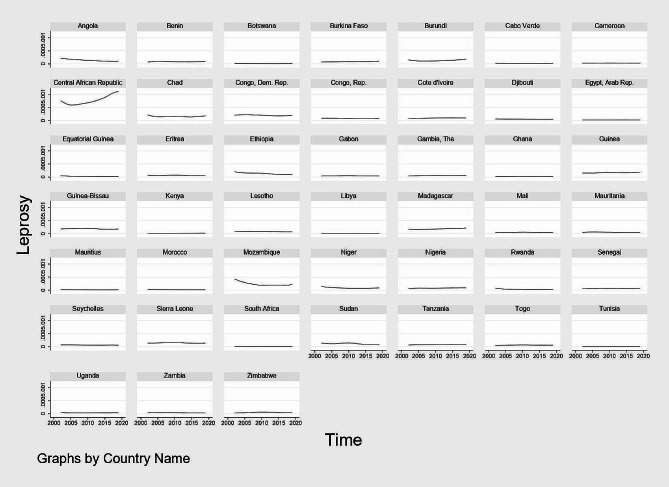
Trend of the prevalence of leprosy per-country, 2002–2019. *Notes* Time is in years

**Fig. 2:**
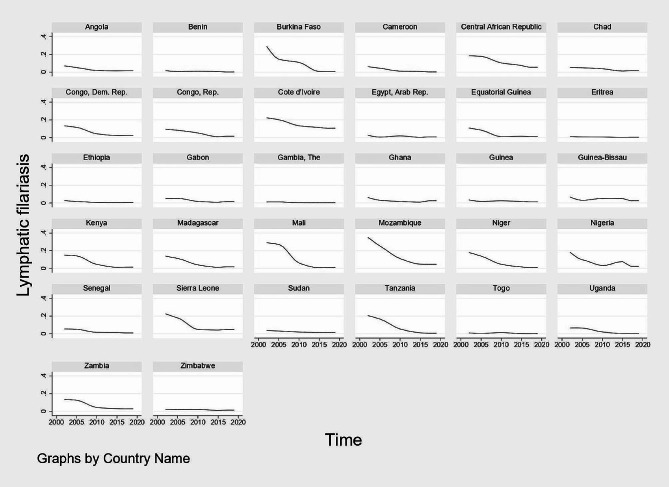
Trend of the prevalence of LF per-country, 2002–2019. *Notes* Time is in years

**Fig. 3:**
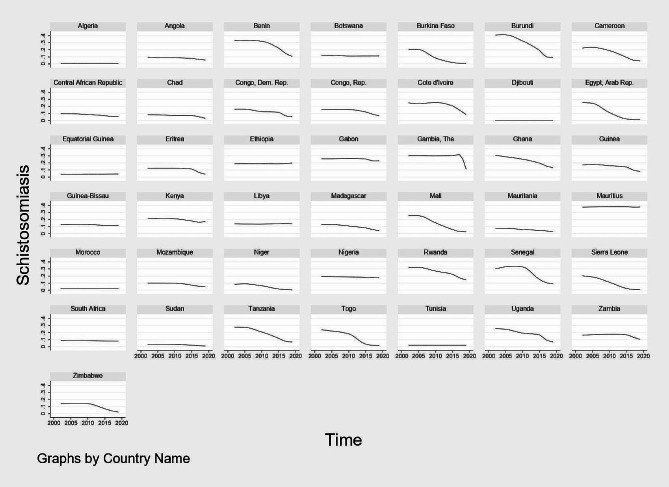
Trend of the prevalence of schistosomiasis per-country, 2002–2019. *Notes* Time is in years

**Fig. 4:**
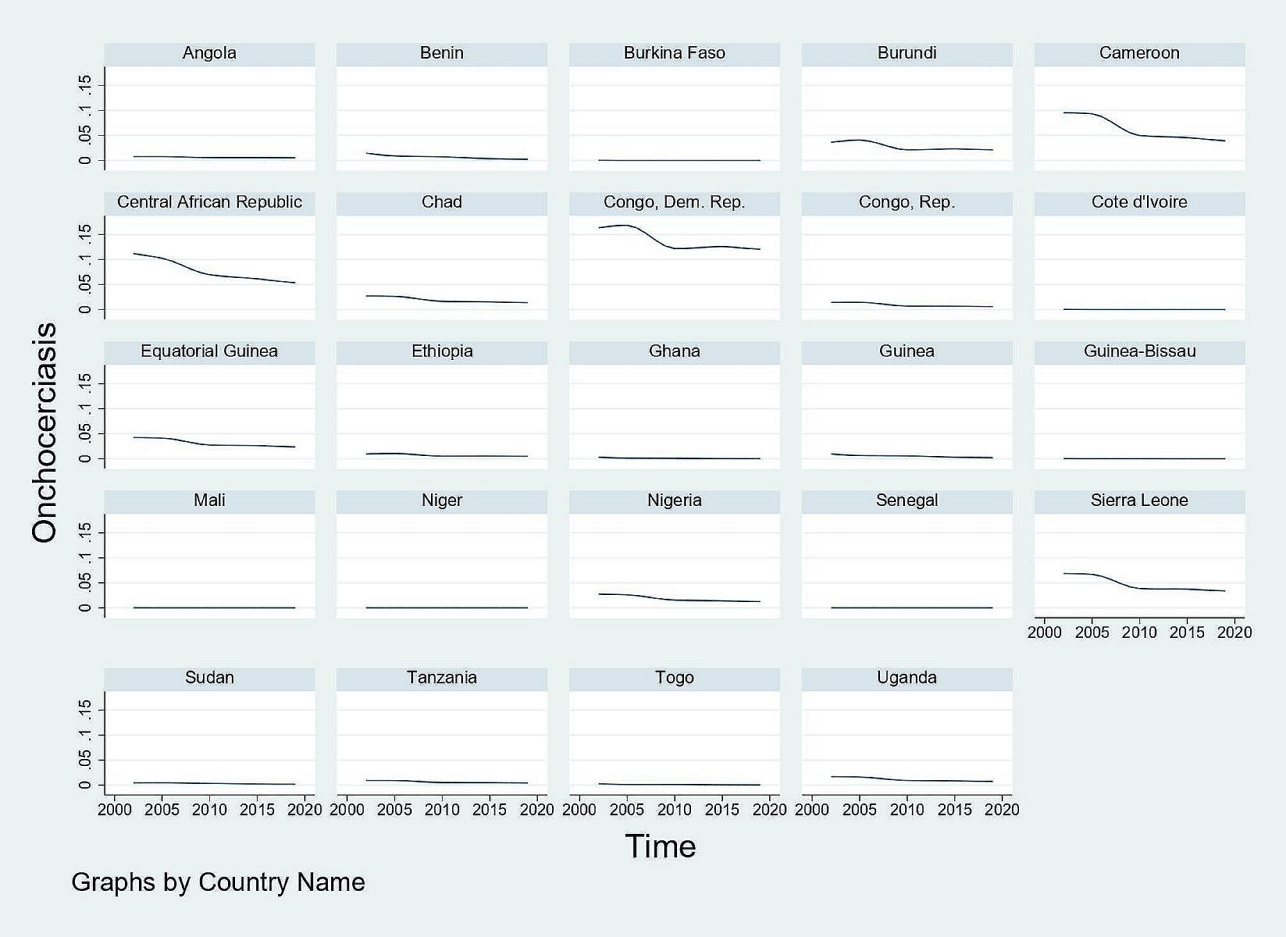
Trend of the prevalence of onchocerciasis per-country, 2002–2019. *Notes* Time is in years

Figures 1, 2, 3 and 4 show graphical trends of the prevalence of NTDs per-country. It can be seen that in the case of leprosy, apart from Central African Republic with an upward trend especially after 2012, majority of the remaining countries show either a downward or a constant trend overtime (Fig. 1). For LF, schistosomiasis and onchocerciasis, no country clearly shows an upward trend while the rest of the countries exhibit either a downward or a constant trend (Figs. 2, 3 and 4).

### Regression estimates of the effects of NTDs on economic performance

This sub-section presents the RE, FE and IVFE regression estimates of the effects of NTDs on economic performance in Africa. All our regression estimates have good fit given the high statistical significance (at 1%) of the overall p-values of our models. The results of the RE (Table 2) and FE (Table 3) regressions are qualitatively similar which confirms the robustness of our estimates. However, since the FE regression estimates are the best based on the test of overidentifying restrictions, we restrict our interpretation to the FE regression estimates.

**Table 2:** RE regression estimates of the effects of NTDs on economic performance in Africa

	(1)	(2)	(3)	(4)
	LogGDP	LogGDP	LogGDP	LogGDP
LogLeprosy	-0.3555^**^			
	(0.1486)			
FDI	-0.0020	-0.0021	-0.0020	-0.0051
	(0.0031)	(0.0030)	(0.0042)	(0.0042)
Inflation	-0.0020^**^	-0.0010^*^	-0.0027^***^	-0.0028^***^
	(0.0010)	(0.0005)	(0.0007)	(0.0004)
Regulatory quality	0.0880	0.2844^***^	0.1404	0.4047^***^
	(0.1254)	(0.0896)	(0.1118)	(0.1025)
LogDomestic investment	0.2923^***^	0.0949^**^	0.1910^***^	0.1419^**^
	(0.0739)	(0.0478)	(0.0614)	(0.0712)
LogExpenditure	-0.0525	-0.1996^**^	-0.0895	-0.0342
	(0.1858)	(0.0931)	(0.1612)	(0.1138)
LogExports	0.1589	0.0796	0.0753	0.1136
	(0.1256)	(0.0804)	(0.0945)	(0.0998)
LogImports	-0.1493	-0.0798	-0.0470	-0.1070
	(0.1198)	(0.0787)	(0.0911)	(0.0945)
LogLymphatic filariasis		-0.2397^***^		
		(0.0181)		
LogSchistosomiasis			-0.2923^***^	
			(0.0303)	
LogOnchocerciasis				-0.2970^***^
				(0.0421)
Constant	19.2377^***^	23.4195^***^	22.6837^***^	21.7435^***^
	(1.7624)	(0.6263)	(0.8486)	(0.6981)
Observations	746	548	734	420
No. of countries	45	32	43	24
Within R^2^	0.1833	0.6820	0.4973	0.6683
Between R^2^	0.1470	0.0363	0.0405	0.0034
Overall R^2^	0.1978	0.0445	0.1033	0.0004
Chi2 stat.	42.1824	309.4314	129.9098	424.4891
Chi2 stat. p-value	0.0000	0.0000	0.0000	0.0000

Cluster robust standard errors in parentheses

^*^p < 0.1, ^**^p < 0.05, ^***^p < 0.01

**Table 3:** FE regression estimates of the effects of NTDs on economic performance in Africa

	(1)	(2)	(3)	(4)
	LogGDP	LogGDP	LogGDP	LogGDP
LogLeprosy	-0.3396^*^			
	(0.1731)			
FDI	-0.0019	-0.0021	-0.0020	-0.0052
	(0.0032)	(0.0031)	(0.0042)	(0.0042)
Inflation	-0.0020^*^	-0.0010^*^	-0.0027^***^	-0.0028^***^
	(0.0010)	(0.0005)	(0.0007)	(0.0004)
Regulatory quality	0.0970	0.2822^***^	0.1379	0.4193^***^
	(0.1307)	(0.0914)	(0.1147)	(0.1039)
LogDomestic investment	0.2889^***^	0.0906^*^	0.1856^***^	0.1071
	(0.0742)	(0.0486)	(0.0620)	(0.0703)
LogExpenditure	-0.0499	-0.2029^**^	-0.0917	-0.0589
	(0.1844)	(0.0939)	(0.1597)	(0.1118)
LogExports	0.1536	0.0738	0.0688	0.0818
	(0.1270)	(0.0811)	(0.0955)	(0.1007)
LogImports	-0.1351	-0.0720	-0.0364	-0.0684
	(0.1217)	(0.0797)	(0.0919)	(0.0959)
LogLymphatic filariasis		-0.2404^***^		
		(0.0182)		
LogSchistosomiasis			-0.2942^***^	
			(0.0309)	
LogOnchocerciasis				-0.3229^***^
				(0.0450)
Constant	19.4677^***^	23.4401^***^	22.7348^***^	21.7402^***^
	(1.9613)	(0.5234)	(0.7963)	(0.6531)
Observations	746	548	734	420
No. of countries	45	32	43	24
Within R^2^	0.1836	0.6821	0.4974	0.6699
Between R^2^	0.1402	0.0340	0.0377	0.0054
Overall R^2^	0.1913	0.0426	0.0997	0.0013
F-stat.	4.7145	37.9422	15.8351	45.1129
F-stat. p-value	0.0003	0.0000	0.0000	0.0000

Cluster robust standard errors in parentheses

^*^p < 0.1, ^**^p < 0.05, ^***^p < 0.01

In the FE estimates, we find that all the selected NTDs have negative statistically significant effects on economic performance. Specifically, a percentage increase in the prevalence of leprosy is found to be associated with a reduction in economic performance by 0.34% at the 10% level of significance. For LF, our findings show that when its prevalence increases by 1%, it is associated with a reduction in economic performance by 0.24% at the 1% level of significance. A 1% increase in the prevalence of schistosomiasis is also found to be associated with 0.29% fall in economic performance while a percentage increase in the prevalence of onchocerciasis is found to be associated with a 0.32% reduction in economic performance, all at the 1% level of significance (Table 3).

With regard to the control variables, we find inflation to exert a negative significant effect on economic performance. Specifically, a unit increase in inflation is found to be associated with a reduction in economic performance by 0.001 units to 0.003 units at either the 1% or 10% level of significance. Nonetheless, per unit (1% in the case of domestic investment) enhancement in regulatory quality and domestic investment is found to be associated with an increase in economic performance by 0.28 units and 0.42 units, and 0.09% to 0.29% respectively, at either the 1% or 10% level of significance. Consumption expenditure is also found to have a negative statistically significant effect on economic performance. Specifically, a percentage increase in consumption expenditure is found to be associated with a fall in economic performance by 0.20% at the 5% level of significance (Table 3).

**Table 4:** IVFE regression estimates of the effects of NTDs on economic performance in Africa

	(1)	(2)	(3)	(4)
	LogGDP	LogGDP	LogGDP	LogGDP
LogLeprosy	-0.4256^**^			
	(0.1882)			
FDI	-0.0017	-0.0014	-0.0009	-0.0034
	(0.0024)	(0.0024)	(0.0031)	(0.0034)
Inflation	-0.0020^*^	-0.0008	-0.0025^***^	-0.0030^***^
	(0.0011)	(0.0006)	(0.0008)	(0.0007)
Regulatory quality	0.1240	0.3059^***^	0.1677	0.3532^***^
	(0.1260)	(0.0985)	(0.1093)	(0.1143)
LogDomestic investment	0.2685^***^	0.0757	0.1732^***^	0.0584
	(0.0707)	(0.0477)	(0.0573)	(0.0691)
LogExpenditure	-0.0305	-0.1953^*^	-0.0699	-0.1158
	(0.1574)	(0.1159)	(0.1416)	(0.1307)
LogExports	0.1359	0.0460	0.0451	0.0206
	(0.1275)	(0.0814)	(0.0818)	(0.0923)
LogImports	-0.1243	-0.0448	-0.0215	-0.0011
	(0.1194)	(0.0813)	(0.0787)	(0.0899)
LogLymphatic filariasis		-0.2413^***^		
		(0.0188)		
LogSchistosomiasis			-0.2770^***^	
			(0.0280)	
LogOnchocerciasis				-0.3552^***^
				(0.0463)
Observations	701	516	692	396
No. of countries	45	32	43	24
F-stat.	4.4564	31.4635	14.9765	18.8212
F-stat. p-value	0.0005	0.0000	0.0000	0.0000
Id stat.	17.4936	15.1991	9.9234	10.9830
Id stat. p-value	0.0002	0.0005	0.0070	0.0041
WI stat.	406.4518	3789.5542	3971.6842	625.9249
Hansen *j* stat.	0.4699	0.2235	0.0484	0.0443
Hansen *j* p-value	0.4930	0.6364	0.8258	0.8333

Cluster robust standard errors in parentheses; WI stat.: Kleibergen-Paap rk Wald F statistic for weak identification; The WI stat. is greater than all the Stock-Yogo critical values (which are for the Cragg-Donald F statistic and i.i.d. errors, and are available upon request); Id stat: Kleibergen-Paap rk LM under-identification test; Hansen *j* stat: Hansen overidentification test

^*^p < 0.1, ^**^p < 0.05, ^***^p < 0.01

For further robustness and to deal with endogeneity, we use the IVFE regression (Table 4) to examine the effects of NTDs on economic performance, and we find the results not to be qualitatively different from the RE and FE regression estimates, especially with regard to the main variables of interest. It must be stressed that our IVFE models do not suffer from under-identification, weak identification and over-identification, which justify the appropriateness of our estimates.

Specifically, using the IVFE estimates, we find a percentage increase in the prevalence of leprosy to be associated with a decrease in economic performance by 0.43% at the 5% level of significance. A percentage increase in the prevalence of LF is also found to be associated with a 0.24% fall in economic performance at the 1% level of significance. Similarly, an increase in the prevalence of schistosomiasis and onchocerciasis is found to be associated with a decrease in economic performance by 0.28% and 0.36%, respectively, at the 1% level of significance. Turning to the control variables, we find that the effects of inflation, regulatory quality, domestic investment and consumption expenditure in the IVFE estimates are qualitatively similar to those found in the RE and FE estimates (Table 4).

## Discussion

In this study, we provide the foremost cross-country macrolevel analysis of the effects of more than one NTD (leprosy, LF, schistosomiasis and onchocerciasis) on economic performance in Africa. As expected, we find an increase in the prevalence of the selected NTDs to be associated with a reduction in economic performance. The results are robust irrespective of the estimation technique used.

Specifically, using the IVFE estimates, a percentage increase in the prevalence of leprosy, LF, schistosomiasis and onchocerciasis is found to be associated with a decrease in economic performance by 0.43%, 0.24%, 0.28% and 0.36% respectively (at either 1% or 5% level of significance). Given the average economic performance figures (see appendix (supplementary material)) of $41.9 billion, $41 billion, $47.2 billion and $36.8 billion among the sampled countries for the leprosy, LF, schistosomiasis and onchocerciasis models, respectively, over the study period, the implication is that, on the average, a percentage increase in the prevalence of leprosy, LF, schistosomiasis and onchocerciasis is associated with a fall in economic performance by $180 million, $98.4 million, $132 million and $133 million^2^, respectively. These findings are not surprising because NTDs are associated with stigma and disabilities that can permanently prevent affected persons from working. In particular, it is not surprising that leprosy (albeit its low prevalence relatively) is associated with the greatest loss in economic performance because it can lead to permanent disability of both the legs and hands. Thus, given the less developed nature of economies in Africa relative to other developed regions, there is the urgent need to deepen measures geared towards tackling these diseases in order to curb the economic losses which could be used for other developmental projects. Specifically, attention should be paid towards improving MDA targeting all at-risk populations as well as enhancing behaviour change, hygiene education and access to improved sanitation and safe water [5]. 

Our findings on the effects of NTDs are in line with some past studies. For instance, in Africa, a study published in 1972 found the yearly loss from schistosomiasis emanating from partial and complete disability to be nearly $446 million [16]. LF has been found to be associated with a productivity cost of $1,023,437 (in thousands) in the WHO African Region [17]. Similarly, LF has been found to be associated with an annual cost (reduced working time and treatment cost) of $842 millon among households and patients in India [41]. A study by Marques et al. [19] found a 0.27% loss in GDP in Western sub-Saharan Africa as a result of blindness or vision impairment, while Kim et al. [18] revealed that, eliminating onchocerciasis in Africa could result in an income gain of $5.9 billion-$6.4 billion. However, caution must be exercised in comparing our findings with those of previous studies because of differences in the study periods and prevalence rates used. In addition, while most of the past studies provided estimates of the total productivity cost associated with these diseases, our study provides the loss in economic performance (GDP) associated with a percentage increase in the prevalence of these diseases.

Turning to the control variables, the results of the negative significant effect of inflation on economic performance could be due to the fact that higher prices can decrease the ability of producers to purchase production inputs, which could reduce economic performance. This outcome concurs with Sakyi and Egyir [21] and Anyanwu [23] who found a negative association between inflation and economic growth among a sample of African countries. Similarly, Boachie [24] found inflation to hamper economic growth in Ghana.

The positive effect of regulatory quality on economic performance is not surprising since an enhancement in regulatory quality can increase private sector development and productivity, which would culminate into enhanced economic performance.

The negative and positive effects of consumption expenditure and domestic investment, respectively, on economic performance are not farfetched because, while consumption serves as a leakage and hence reduces productive capacity, investment serves as an injection, which increases productive capacity, hence, economic performance [22]. Our finding on domestic investment is in tandem with Ibrahim and Alagidede [42], Egyir et al. [25], and Oyebowale and Algarhi [43] who found investment to enhance economic growth in Africa.

Notwithstanding, the study does not find out whether the effects of the selected NTDs on economic performance, significantly differs among males and females. Also, this study does not cover NTDs such as trachoma and intestinal worms that are also found on the African continent. We therefore suggest that, future studies look into these issues.

## Conclusion

NTDs such as leprosy, LF, schistosomiasis and onchocerciasis affect several people on the African continent. Aside from severe morbidity, these NTDs are associated with permanent disabilities which can reduce the economic productivity of affected people, resulting into lower economic performance at the macrolevel. Nonetheless, cross-country empirical evidence of the effects of these NTDs on economic performance at the macrolevel in Africa is sparse. To this end, this study examines the effects of leprosy, LF, schistosomiasis and onchocerciasis on economic performance at the macrolevel in 24 to 45 African countries (depending on the NTD in question) for the period, 2002–2019. We find that a percentage increase in the prevalence of leprosy, LF, schistosomiasis and onchocerciasis is associated with a fall in economic performance by 0.43%, 0.24%, 0.28% and 0.36% respectively (at either 1% or 5% level of significance). These translate into respective economic losses of $180 million, $98.4 million, $132 million and $133 million per a percentage increase in the prevalence of these diseases. There is, therefore, the need to bolster integrated efforts towards tackling these diseases in order to curb their deleterious effects on economic performance. Such efforts should include improving MDA targeting all at-risk populations as well as enhancing behaviour change, hygiene education and, access to improved sanitation and safe water [5]. 

### Electronic supplementary material

Below is the link to the electronic supplementary material.

Supplementary Material 1

## Data Availability

The data employed by this study are available to the public for free from the websites of the World Bank (https://databank.worldbank.org/reports.aspx?source=World-Development-Indicators#advancedDownloadOptions; https://databank.worldbank.org/source/worldwide-governance-indicators#advancedDownloadOptions) and the Global Burden of Diseases Study (https://vizhub.healthdata.org/gbd-results/). The data used for this study are aggregated (at the macrolevel), hence, do not contain information on any identifiable human subjects. All the approaches used in this study conform to the relevant regulations and guidelines.

## Footnotes

1 This study is added beause blindness and vision impairment could be caused by onchocerciasis. It should also be noted that, since vision impairment can lead to blindness, we do not regard them as different diseases.  

2 It should be noted that the average GDP figures in the supplementary file and the respective coefficients are used in arriving at these figures.
