# Supplementary material for: The effects of selected neglected tropical diseases on economic performance at the macrolevel in Africa
Source: BMC Infect Dis. 2024 May 2;24:462. doi: 10.1186/s12879-024-09302-3 (PMC11064357; doi:10.1186/s12879-024-09302-3)
Supplement: Supplementary file 1 — Supplementary Material 1 [file 12879_2024_9302_MOESM1_ESM.docx]

**Descriptive statistics of variables for countries used in the leprosy models**

| Variable | Obs | Mean | Std. Dev. | Min | Max |
| --- | --- | --- | --- | --- | --- |
| GDP | 791 | 4.189e+10 | 8.332e+10 | 7.019e+08 | 5.094e+11 |
| Leprosy | 810 | .00009 | .0001215 | 3.80e-06 | .001 |
| FDI | 802 | 4.127 | 5.817 | -11.197 | 57.877 |
| Inflation | 790 | 8.48 | 16.968 | -24.847 | 225.395 |
| Regulatory quality | 810 | -.674 | .613 | -2.282 | 1.197 |
| Domestic investment | 747 | 22.444 | 9.065 | 2 | 81.021 |
| Expenditure | 759 | 83.835 | 17.444 | 16.713 | 145.517 |
| Exports | 774 | 32.306 | 21.772 | .611 | 166.718 |
| Imports | 774 | 39.724 | 21.663 | .588 | 191.458 |
|  | | | | | |

**Descriptive statistics of variables for countries used in the LF models**

| Variable | Obs | Mean | Std. Dev. | Min | Max |
| --- | --- | --- | --- | --- | --- |
| GDP | 568 | 4.102e+10 | 8.280e+10 | 7.019e+08 | 5.094e+11 |
| Lymphatic filariasis | 576 | .05 | .059 | .001 | .351 |
| FDI | 568 | 4.025 | 5.777 | -11.197 | 46.275 |
| Inflation | 568 | 9.59 | 19.404 | -21.165 | 225.395 |
| Regulatory quality | 576 | -.839 | .488 | -2.227 | .099 |
| Domestic investment | 548 | 21.813 | 9.324 | 2 | 81.021 |
| Expenditure | 548 | 83.737 | 17.53 | 16.713 | 145.517 |
| Exports | 556 | 27.67 | 15.774 | .611 | 89.224 |
| Imports | 556 | 34.02 | 13.492 | .588 | 113.661 |
|  | | | | | |

**Descriptive statistics of variables for countries used in the schistosomiasis models**

| Variable | Obs | Mean | Std. Dev. | Min | Max |
| --- | --- | --- | --- | --- | --- |
| GDP | 755 | 4.722e+10 | 8.598e+10 | 7.019e+08 | 5.094e+11 |
| Schistosomiasis | 774 | .141 | .098 | .002 | .413 |
| FDI | 766 | 3.78 | 5.425 | -11.197 | 46.275 |
| Inflation | 754 | 8.654 | 17.275 | -24.847 | 225.395 |
| Regulatory quality | 774 | -.711 | .613 | -2.282 | 1.197 |
| Domestic investment | 735 | 22.32 | 9.033 | 2 | 81.021 |
| Expenditure | 735 | 82.487 | 17.401 | 16.713 | 145.517 |
| Exports | 743 | 30.543 | 19.966 | .611 | 166.718 |
| Imports | 743 | 36.299 | 17.335 | .588 | 191.458 |
|  | | | | | |

**Descriptive statistics of variables for countries used in the onchocerciasis models**

| Variable | Obs | Mean | Std. Dev. | Min | Max |
| --- | --- | --- | --- | --- | --- |
| GDP | 432 | 3.683e+10 | 7.838e+10 | 7.019e+08 | 5.094e+11 |
| Onchocerciasis | 432 | .02 | .033 | 0 | .168 |
| FDI | 432 | 3.556 | 5.539 | -11.197 | 46.275 |
| Inflation | 432 | 8.819 | 15.884 | -21.165 | 196.984 |
| Regulatory quality | 432 | -.858 | .414 | -1.705 | .099 |
| Domestic investment | 420 | 22.243 | 9.689 | 3.949 | 81.021 |
| Expenditure | 420 | 83.427 | 16.78 | 16.713 | 140.815 |
| Exports | 420 | 26.815 | 16.912 | .611 | 89.224 |
| Imports | 420 | 33.132 | 13.654 | .588 | 113.661 |
|  | | | | | |
